# Enterovirus RNA in Peripheral Blood May Be Associated with the Variants of rs1990760, a Common Type 1 Diabetes Associated Polymorphism in *IFIH1*

**DOI:** 10.1371/journal.pone.0048409

**Published:** 2012-11-07

**Authors:** Ondrej Cinek, German Tapia, Elisabet Witsø, Lenka Kramna, Katerina Holkova, Trond Rasmussen, Lars C. Stene, Kjersti S. Rønningen

**Affiliations:** 1 2nd Faculty of Medicine, Department of Paediatrics, Charles University in Prague and University Hospital Motol, Prague, The Czech Republic; 2 Division of Epidemiology, Norwegian Institute of Public Health, Oslo, Norway; 3 Oslo University Hospital, Rikshospitalet, Oslo, Norway; La Jolla Institute for Allergy and Immunology, United States of America

## Abstract

**Objective:**

Polymorphisms in the *IFIH1* (common rs1990760 and four rare rs35667974, rs35337543, rs35744605, rs35732034) have been convincingly associated with type 1 diabetes. The encoded protein (interferon-induced helicase C domain-containing protein 1) senses double-stranded RNA during replication of *Picornavirales*, including *Enterovirus*, a genus suspected in the etiology of type 1 diabetes. We therefore investigated whether the polymorphisms are associated with differences in the frequency of enterovirus RNA in blood.

**Research Design and Methods:**

The study included 1001 blood samples, each from a child participating in the Norwegian ‘Environmental Triggers of Type 1 Diabetes: the MIDIA study’. The enterovirus RNA was tested using qualitative semi-nested real-time reverse transcriptase PCR on RNA extracted from frozen cell packs after removal of plasma. Stool samples previously analyzed for enterovirus RNA were available in 417 children.

**Results:**

The genotypes of *IFIH1* rs1990760 were associated with different frequencies of enterovirus RNA in blood (7.0%, 14.4% and 9.5% bloods were enterovirus positive among children carrying the Ala/Ala, Ala/Thr and Thr/Thr genotypes, respectively, p = 0.012). This association remained essentially unchanged after adjustment for age and calendar year. The presence of enterovirus in the concomitantly sampled stool further increased the likelihood of enterovirus RNA in blood (odds ratio 2.40, CI 95% 1.13–4.70), but did not affect the association with *IFIH1* rs1990760. The rare polymorphisms (individually, or pooled) were not significantly associated with enterovirus RNA in blood.

**Conclusions:**

The common *IFIH1* SNP may modify the frequency of enterovirus RNA in blood of healthy children. This effect can help explain the association of *IFIH1* with type 1 diabetes.

## Introduction

Enteroviruses, namely the members of the species *Human enterovirus A - D* (genus *Enterovirus*, family *Picornaviridae*, order *Picornavirales*), have been suspected from involvement in the etiology and pathogenesis of type 1 diabetes (T1D), reviewed e.g. in [1]. Enterovirus positivity has been observed in the pancreatic tissues and gut mucosa at T1D onset more often than in matched controls [2]–[4], although its presence at disease onset may be a consequence rather than a cause of the autoimmune process. Reports are inconclusive as to the actual ability of enterovirus to *initiate* the autoimmune process leading to T1D: enterovirus can predict a small fraction of the cases with autoimmune process in Finnish studies [5]–[8], while no association with autoimmunity was found in other populations [9], [10].

Recently, substantial support to the role of enterovirus in T1D pathogenesis was provided by the discovery of an association between the *IFIH1* polymorphisms and T1D. The *IFIH1* gene encodes for the interferon-induced helicase C domain-containing protein 1 which acts as a sensor of long intracellular double stranded RNA produced during replication of certain viruses, including *Picornaviridae* [11], [12]. It is essential for activating antiviral responses, in particular the type I interferons (reviewed in [13]). Its association with T1D was first observed in a genome-wide scan where the common SNP rs1990760 had its minor allele (guanine, encoding the amino acid alanine at the position 946) associated with a slightly decreased risk of T1D as compared to the major allele (adenine, encoding the amino acid threonine) [14]. It was then confirmed in several studies, and similar association has been detected also in other autoimmune disorders (e.g. multiple sclerosis [15], [16] and Graves' disease [17]). The causal role of *IFIH1* was later reinforced by the discovery of four its rare variants associated with T1D [18].

The mechanisms by which the *IFIH1* polymorphisms modify the risk of T1D and other autoimmune diseases are presently unknown, but most works agree that the minor alleles (protective from T1D) are associated with diminished transcription or reduced function of the *IFIH1* [19]–[22]. Conceivably, the *IFIH1* polymorphisms might influence not only the intensity of inflammation following enterovirus infection, but also the frequency and timing of enterovirus infection, and thus modify the likelihood of prediabetic autoimmunity. This is a testable hypothesis: a weaker immune response to enterovirus (associated with the minor alleles) should be mirrored by increased replication of enterovirus, detectable either as its higher frequency, or higher viral load. In our earlier study where we studied enterovirus in faeces, one of the rare variants (rs35732034, a mutation in the conserved splice site of exon 14) was weakly associated with enterovirus in the faeces [23].

In the present study, we aimed to investigate whether the *IFIH1* polymorphisms are related to the frequency of enterovirus RNA in blood samples obtained from healthy children. We expected that the findings in blood, as compared to our previous studies of gut infection, should better reflect the putative effect of *IFIH1* on the course of infection: positivity for enterovirus in blood means that the virus has passed through immunological barriers, including the ones where *IFIH1* acts.

## Materials and Methods

### Study subjects

The subjects were healthy children from the MIDIA study (a Norwegian acronym for “Environmental Triggers of Type 1 Diabetes”). Babies were recruited from the general Norwegian population at public health centers throughout the country [24]. During 2001–2007, 46,939 newborns were genotyped to identify HLA DRB1*04:01-DQA1*03-DQB1*03:02/DRB1*03-DQA1*05-DQB1*02 (DR4-DQ8/DR3-DQ2), the HLA genotype conferring the highest risk of T1D. The resulting high-risk cohort has been followed for development of autoimmunity and T1D. Furthermore, several hundred children with other genotypes were included in order to investigate the effect of HLA on the frequency of enterovirus infection in the gut [25].

In the current analysis, one blood sample and up to two concomitantly obtained stool samples were analyzed from each of 1,001 subjects (512 females, 489 males): 646 carrying the highest-risk genotype and 355 carrying other HLA genotypes. The median age at the blood sample collection was 12.3 months (IQR 9.2–13.5 months); the samples were taken over the period of 2004–2009, most often in 2005, 2006 and 2007 (27%, 34% and 20% respectively).

### Ethics Statement

The parents consented in writing on behalf of the participants involved in the study (babies and infants recruited shortly after birth). The study, including the consent procedure, was approved by The Regional Committee for Medical Research Ethics (IRB name: “Regional Med Resch Ethics Comm South IRB #2 - South-East A”, IRB00001871) and the Norwegian Data Inspectorate.

### Blood sample handling and nucleic acid extraction

Capillary blood was taken into an EDTA-anticoagulated tube (0.3–1 ml), centrifuged and plasma transferred to another tube, while the cell pack was stored frozen in the original tube at −80°C until further processing. From the cell sediments, DNA and RNA were co-purified using a modified procedure for the QIAamp DNA Blood BioRobot MDx Kit (Qiagen, Hilden, Germany). The cell pack was thawed in a refrigerated shaking incubator at +8°C, 300 rpm, adjusted with refrigerated normal saline to approximately 300 µl and added to 50 µl of Qiagen Proteinase prefilled in a deep-well plate kept on ice. Then, 300 µl of Qiagen AL buffer was added and mixed and the extraction proceeded further according to the manufacturer's instructions. Negative control (normal saline with diluted West Nile Virus Armored RNA, Asuragen, USA) was present in 7–9 positions in every 96-well plate, scattered in irregular patterns. The quantity of DNA in the extract was checked by determining the copy number of human *ALB* gene, whereas the cellular RNA content was assessed using reverse transcription real-time PCR for the human *B2M* or *GUSB* transcripts. Of the 1001 extracts, 25 had a very low or no detectable level of human mRNAs, which may have reflected a low cell content of the starting material caused by too small amount of the capillary sample, or a failure of the extraction process.

### Detection of enterovirus RNA in nucleic acid from blood

Enterovirus RNA was tested using a semi-nested assay. The first round was performed as a one-tube reverse transcription PCR with the Quantitect Probe RT-PCR kit (Qiagen) in a duplicate at a total volume of 15 µl, consisting of 1× Quantitect PCR Master Mix, 900 nM ENT-R (5′-ATTGTCACCATAAGCAGCCA-3′), 300 nM ENT-F (5′-CCCTGAATGCGGCTAATCC-3′) primers, 150 nM ENT-probe (FAM-5′-AACCGACTACTTTGGGTGTCCGTGTTTC-3′-TAMRA; the primers are probes in the first round were taken from [26]), 1× QuantiTect RT Mix and 2 µl template nucleic acid extract. The PCR run for 45 cycles with a thermal profile according to the manufacturer's instructions. Two µl PCR product from each replicate were then mixed into 800 µl sterile PCR water in a deep-well plate and used as a template in the second PCR round. This second round was done using the AmpliTaq Gold chemistry (Applied Biosystems, Foster City, CA) in a total volume of 15 µl containing 1× PCR buffer, 200 uM each dNTP, 500 nM of each primer ENT-PolF (5′- CGTAACGCGCAAGTCTGTGG-3′), ENT-nonP (5′- GTCGTAATGGGYAACTCYGCAGCG-3′), ENT-R, 150 nM ENT-probe, 0.4 U AmpliTaq Gold polymerase and 2 µl of the diluted first-round product as a template. The thermal profile had 25 cycles. Along with the samples, a standard curve was run, constructed using serial six-fold dilutions of Armored RNA Enterovirus (Ambion, USA); the standard curve (spanning 375000 copies to a theoretical 1 copy per µl template) controlled the PCR efficiency in the first round, but lost its quantitative character in the second round because its first points had reached the PCR plateau already in the first round. Both rounds were run on a LightCycler 480 (Roche, Mannheim, Germany) equipped with a 384-well thermal block. Neither of the negative controls employed in the two rounds of PCR showed any signal. To further safeguard against cross-contamination, we verified that the enterovirus PCR products were diverse by using separate amplification in the second round with the ENT-PolF or ENT-nonP primers, and we attempted sequencing of these short products with the BigDye terminator 1.1 chemistry (Applied Biosystems, USA) which also showed a certain degree of diversity.

### Stool samples

Stool samples were collected from all individuals in the study. However, some of the analyzed blood samples were collected beyond the date of the last available stool sample and some stools have not been yet tested. Therefore data on enterovirus in the concomitantly collected stool samples (time window spanning 30 days before to 15 days after collection of blood sample) are available only in a subset of 417 children. Of these 417 subjects who had at least one such sample available, 322 had one and 95 had two samples tested within the time window. Enterovirus RNA quantities in the stool samples were assayed previously using a single tube reverse transcription PCR [23], [25].

### IFIH1 genotyping

We genotyped the common *IFIH1* SNP rs1990760 polymorphism (exon 15, A946T) using a TaqMan SNP assay (Applied Biosystems) and the minor *IFIH1* polymorphisms (rs35667974, rs35337543, rs35744605, rs35732034) with the MassARRAY system using iPLEX chemistry (Sequenom, San Diego, CA), as reported previously [23]. All genotypes were in Hardy-Weinberg equilibrium. The genotype frequencies found for the common *IFIH1* rs1990760 polymorphism were Ala/Ala 15.7%, Ala/Thr 48.5%, Thr/Thr 35.8%; the minor allele frequencies of rs35667974, rs35337543, rs35744605 and rs35732034 were 2.9%, 0.6%, 0.7% and 0.6%, respectively. All carriers of the rare variants were heterozygous, neither carried more than one rare variant, and in total 9.6% of the subjects carried at least one minor allele of these four rare polymorphisms. After we detected an association of rs1990760 with enterovirus in blood, we further genotyped the rs3747517 (exon 13, H843R) using a TaqMan SNP assay.

### Statistical analysis

The frequency of enterovirus RNA in blood was first assessed in univariate analyses against *IFIH1* genotypes, gender, year of sampling, seasonality, age at blood sample, HLA risk of T1D and concomitant stool positivity for enterovirus. The variables associated therein were then entered into a multivariable regression model along with the level of HLA risk (highest T1D risk versus all other genotypes; the reason was its role in the study design, because the HLA risk was connected to a difference in the length of observation with stool samples, although by itself it was not associated with the risk of enterovirus RNA in blood). The dependent variable was dichotomous: blood positive or negative in the seminested enterovirus PCR. Two models were employed: the first included all blood samples but lacked the stool enterovirus as the independent variable; the second had concomitant stool positivity as one of the predictors and thus was limited to the 417 subjects where such stool samples were available.

A sensitivity analysis was performed for stool positivity as the predictor: the original time span of 30 days before to 15 days after the blood samples was extended to 60 days and then 90 days. Also, we assessed the effect of excluding 25 samples with little or no detectable human RNA, and of 12 children who developed islet autoantibodies indicative of ongoing islet autoimmunity.

### Sample size and power considerations

Based on our available sample (N = 1000), information available from previous studies [14], [18] on genotype frequencies for the common and four rare IFHI1 SNPs, and assumption of an overall enterovirus positivity rate of 10%, we calculated how large the true relative risk had to be in order for our study to have approximately 70–80% power or more to detect a significant association. Using the program Power [27] we found that if the true relative risk was 2.0, we had 77% power to detect a significant difference between carrying any of the four rare variants vs. none. By further assuming a model of multiplicative allelic effect for rs1990760, the estimated power was 75% to find an overall significant association if the true relative risk was 2.0 comparing the two homozygote genotypes (Thr/Thr vs. Ala/Ala).

## Results

Enterovirus RNA was present in 115/1001 blood samples (11.5%, 95% CI 9.5%–13.5%). The presence of enterovirus RNA in the blood was significantly more frequent in children carrying the rs1990760 *IFIH1* Ala/Thr genotype as compared to the other genotypes (**Table 1**). The four other, rare *IFIH1* variants were not significantly associated with the enterovirus RNA in bloods. The second common coding SNP in *IFIH1*, rs3747517 (R843H in exon 13), which was genotyped after the association with rs1990760 had been observed, was not found associated with enterovirus positivity in univariate analysis (p = 0.063), and did not improve the multivariable regression models' fit.

**Table 1:** Factors tested for association with enterovirus positivity in the univariate analyses.

	Enterovirus RNA in blood, frequency % in the category (n with viremia/n subjects)	Odds ratio [95% CI] for enterovirus RNA in blood (univariate analysis)
**All subjects**	11.5% (115/1001)	
***IFIH1*** ** rs1990760** (1)		
subjects with **Ala/Ala** genotype	7.01% (11/157)	*1.00, reference*
subjects with **Ala/Thr** genotype	14.4% (70/486)	2.23 [1.15–4.33], p = 0.018
subjects with **Thr/Thr** genotype	9.50% (34/358)	1.39 [0.69–2.83], p = 0.359
**Rare variants in the ** ***IFIH1*** (2)		
subjects with **wild-type variants at all four** SNPs	11.4% (103/903)	*1.00, reference*
subjects with the **rare variant** positive at any of the SNPs	12.5% (12/96)	1.11 [0.59–2.10], p = 0.750
**Gender**		
Female	11.5% (59/512)	*1.00, reference*
Male	11.5% (56/489)	0.99 [0.67–1.46], p = 0.972
**Seasonality**. Month of sample, Walter and Elwood test: p = 0.41		
Jan–Mar	9.1% (24/264)	
Apr–Jun	13.3% (37/278)	
Jul–Sep	12.1% (29/240)	
Oct–Dec	11.4% (25/219)	
**Year of sampling** (3)		
2004–2005	15.3% (52/340)	*1.00, reference*
2006	11.5% (39/340)	0.72 [0.45–1.12], p = 0.144
2007–2009	7.5% (24/321)	0.45 [0.27–0.75], p = 0.002
**Age at blood sample**		
0–5.9 months	19.5% (23/118)	*1.00, reference*
6–11.9 months	10.4% (26/251)	0.48 [0.26–0.88], p = 0.018
12–17.9 months	13.4% (57/424)	0.64 [0.38–1.09], p = 0.103
18+ months	4.3% (9/208)	0.19 [0.08–0.42], p<10^−3^
**HLA risk for T1D** (subset aged 10–14 mo)(4)		
high-risk	12.5% (16/128)	*1.00, reference*
children with other genotypes	15.3% (54/353)	1.26 [0.69–2.30], p = 0.443
**Enterovirus** positivity in stool taken within 30 days before to 15 days after the blood sampling date(5); subset of 417 blood samples		
**none** of the concomitant stool samples positive	13.5% (47/349)	*1.00, reference*
**one or more** concomitant stool samples positive	25.0% (17/68)	2.14 [1.14–4.02], p = 0.018

(1) *IFIH1* 946Ala is associated with a decrease, whereas the 946Thr with an increase in T1D risk in most studies [37]. Having detected the association of rs1990760 heterozygosity with an increased risk of enterovirus in blood, we further complemented the genotyping with the rs3747517 SNP which is in linkage disequilibrium with the rs1990760 (D′ = 1.0, r^2^ = 0.59 in our study, calculated according to [38]): however, the rs3747517 polymorphism was not significantly associated with enterovirus positivity (P = 0.063), and did not improve the fit of the multivariable analyses.

(2) The tested SNPs were rs35667974, rs35337543, rs35744605, rs35732034. Their minor alleles have been found associated with a decreased risk of T1D [18].

(3) The proportion of *IFIH1* genotypes was not different among the years of sampling (p = 0.43).

(4) The analysis of HLA was restricted to a subset of sample taken at age 10–14 months, as the sampling schedules differed profoundly between the high-risk carriers and the non high-risk individuals. Whilst the former had a more even distribution of samples, the latter was almost exclusively restricted to the age 10–14 months and taken in 2005–2006 only. High risk, the genotype DRB1*04:01-DQA1*03-DQB1*03:02/DRB1*03-DQA1*05-DQB1*02 (DR4-DQ8/DR3-DQ2).

(5) Stools from intervals more distant to the blood sample were not associated (the lowest p = 0.12 for interval 31 to 60 days before blood).

The proportion of positive blood samples depended on the year of sampling, on the age of the subject and on the presence of enterovirus in concomitantly collected stool, but there was no significant association with gender, or season of sample collection. In a multivariable regression model with predictors identified from the univariate analysis, the association between the *IFIH1* rs1990760 genotype and enterovirus RNA in blood remained almost unchanged (**Table 2** **, left column**).

**Table 2:** The *IFIH1*-associated risk in a multivariable analysis.

	Odds ratio [95% CI], p, in a model *without* the stool positivity as predictor (1001 blood samples)	Odds ratio [95% CI], p, in a subset of samples where a concomitant stool was available(1) (417 blood samples)
***IFIH1*** ** rs1990760** (2)		
Ala/Ala	*1.00, reference*	*1.00, reference*
Ala/Thr	2.18 [1.12–4.27], p = 0.022	2.23 [0.93–5.32], p = 0.072
Thr/Thr	1.37 [0.67–2.82], p = 0.380	1.50 [0.59–3.79], p = 0.394
**Stool** -**30 days before to 15 days after the blood**		
all negative	(not included in the model)	*1.00, reference*
one or more positive	(not included in the model)	2.40 [1.13–4.70], p = 0.010
**Year of sampling**		
2004–2005	*1.00, reference*	*1.00, reference*
2006	0.68 [0.43–1.07], p = 0.099	0.58 [0.32–1.04], p = 0.069
2007–2009	0.63 [0.34–1.12], p = 0.116	0.66 [0.08–5.24], p = 0.691
**Age at blood sample**		
0–5.9 months	*1.00, reference*	*1.00, reference*
6–11.9 months	0.44 [0.23–0.82], p = 0.011	0.37 [0.15–0.93], p = 0.035
12–17.9 months	0.41 [0.20–0.84], p = 0.015	0.33 [0.11–0.98], p = 0.047
18+ months	0.20 [0.09–0.45], p<10^−3^	0.32 [0.04–2.69], p = 0.292
**HLA risk for T1D**		
high-risk(3)	*1.00, reference*	*1.00, reference*
other genotypes	1.67 [0.88–3.15], p = 0.114	1.40 [0.60–3.27], p = 0.440

(1) A stool sample tested for enterovirus, collected within the interval from 30 days before to 15 days after the blood: the dataset was restricted to 417 bloods having at least one such tested stool available.

(2) Adding the terms for the rs3747517 did not improve the fit of the model.

(3) DRB1*04:01-DQA1*03-DQB1*03:02/DRB1*03-DQA1*05-DQB1*02 (DR4-DQ8/DR3-DQ2).

The magnitude of the observed *IFIH1* association remained essentially unchanged also after restriction to the subset of 417 children that had a concomitant stool sample tested for enterovirus RNA (**Table 2** **, right column**; the statistical significance diminished due to the decreased sample size). The presence of enterovirus RNA in concomitant stool samples was associated with a significant, approximately two-fold higher frequency of enterovirus RNA in the blood. The effect of a concomitant stool positivity dissipated with extending the time span over which the stool samples were collected relative to the date of blood sample: if longer intervals than a month before the blood samples were taken as predictors, no effect was observed.

No changes in the results were observed after exclusion of the 25 samples that contained only little or no human RNA (indicating low content of cells in the sample), or of the 12 children who developed islet autoimmunity up to the date of blood sampling.

## Discussion

### IFIH1 genotypes and frequency of enterovirus RNA in blood

The genotypes of the common *IFIH1* SNP rs1990760 were significantly associated with different levels of risk of enterovirus RNA in the peripheral blood, the highest frequency being observed among the Ala/Thr heterozygotes. Although our material is probably not optimal to determine an exact genetic model, the observation resembles heterosis in classical genetics. Molecular heterosis has been repeatedly noted in humans (reviewed in [28]). Up to our best knowledge, molecular heterosis has been observed in *IFIH1* only once: in a Belgian case-control association study, the heterozygous genotype of rs1990760 was associated with significant protection from T1D relative to both homozygotes [29]. Although such a result would point in the same direction as our data (decreased immune response in the heterozygotes), it appears to us that the underlying reason for their result may have been an artefact due to an undetected deviation from the Hardy-Weinberg equilibrium in the control dataset (p = 0.002 for the deviation upon recalculation from the published data).

We did not find any association between the four rare T1D-associated *IFIH1* variants and the enterovirus RNA in blood, which is potentially attributable also to a lower statistical power due to the low frequency of these alleles.

### Potential functional explanations

Several possible explanations exist for an observation of molecular heterosis [28]. An effect of the rs1990760 on transcription levels seems unlikely: a study utilizing an accurate and sensitive technology was unable to find differences in expression between its two alleles [30], and the authors proposed that the common *IFIH1* polymorphisms may have other unknown functions, or act through changes in the tertiary structure of the protein. Since the IFIH1 protein homodimerizes and oligomerizes upon its binding to dsRNA [31], and as the rs1990760 encodes an amino acid in a region important for dimerization of the RIG-I-like proteins [32], we may speculate that a dimer consisting of two different protein variants may assemble less readily or act less effectively as compared to homodimers consisting of identical variants. Indeed, in dimeric proteins, such a mechanism is often proposed as an explanation of molecular heterosis [28].

### Comparison to previous studies on IFIH1

Chistiakov *et al* [22] investigated the effects of two of the rare substitutions (rs35744605, rs35667974) on the enterovirus frequency in the Russian population: the minor allele of the latter SNP (an allele protective from T1D) was associated with a clear and significant *decrease* in the enterovirus-positive proportion of RNA serum in T1D patients – a relation also rather difficult to explain. The authors did not investigate the rs1990760. One more study marginally touched the topic of *IFIH1* related risk of enterovirus RNA in blood, but the sample size was far too small for efficient association testing [33].

In our previous study performed on the fecal material, we did not observe associations of enterovirus with the rs1990760, whereas we noted a borderline-significant increase in enterovirus frequency in carriers of one of the rare loss-of-function polymorphisms, the rs35732034 [23]. The influence of the immunity (and thus of *IFIH1*) on enterovirus replicating in the gut may be indeed less pronounced than in the blood – it is assumed that the antibodies can prevent spreading of the infection from the gut, but cannot provide “sterilizing immunity” [34]. The difference in *IFIH1* effect between blood and stool is in line also with the current evidence linking enterovirus to islet autoimmunity (or T1D) more strongly for enterovirus RNA detected in blood than for fecal enteroviral RNA [35].

### Relation between stool and blood positivity for enterovirus

**Figure 1:**
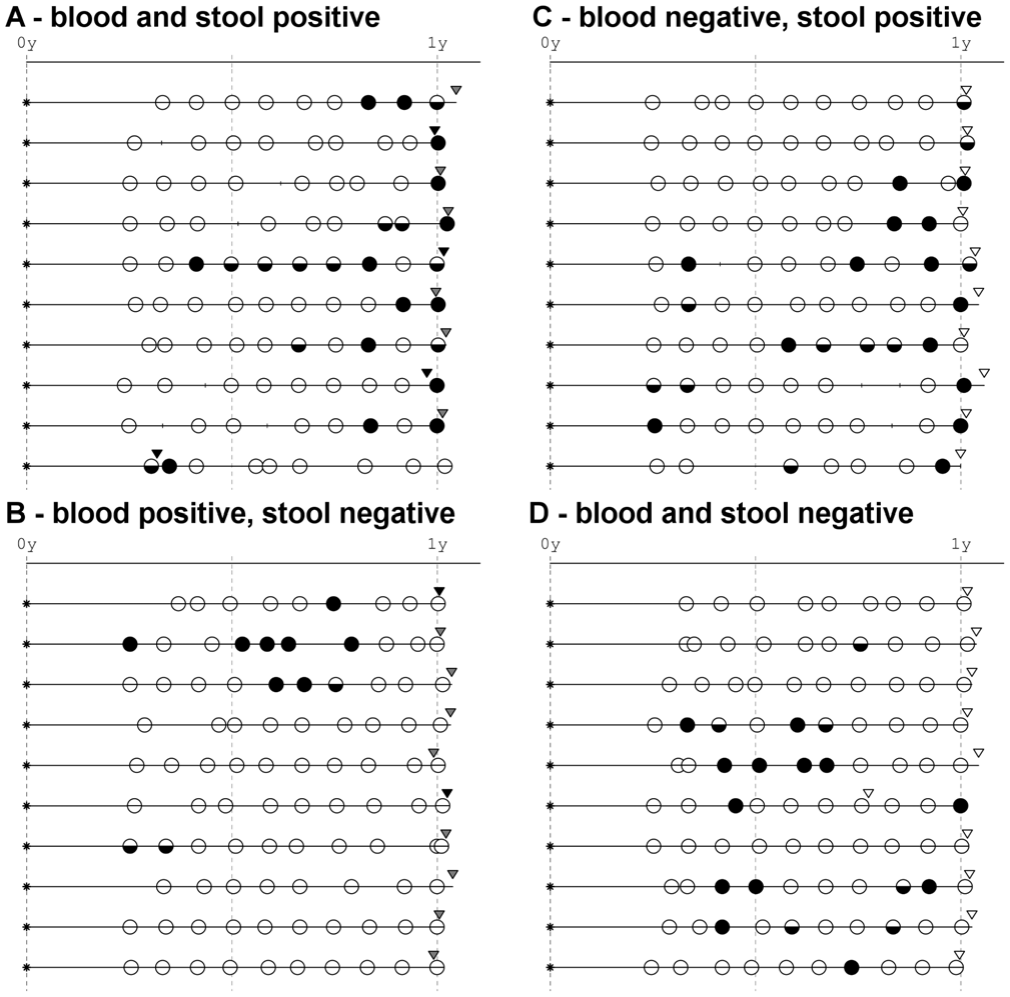
Examples of relation between enterovirus RNA in blood and in the concomitant stool samples. A. The tested blood, and the concomitant stool were both positive. B. The tested blood was positive, while the concomitant stool was negative. C. The tested blood was negative, while the concomitant stool was positive. D. The tested blood, and the concomitant stool were both negative. The four possible combinations of positivity and negativity of stool and blood are shown, each exemplified by ten individuals. Typical situations are shown. *Concomitant* is a stool sample taken within the interval 30 days before to 15 days after the tested blood. The graphs show the life lines of the infants during their first year of life. Each line denotes one child. The children are aligned by birth (asterisk), the horizontal axis shows age (yrs). The triangles are blood samples: full black, positive already in the first PCR round; dark grey, positive in the second round of PCR; empty, negative. The circles correspond to the stool samples: full circles, stool with more than 10 000 enterovirus copies per µl RNA; lower half-circles, stool positive up to 10 000 copies per µl RNA; empty circles, stool negative or with a trace of enterovirus up to 10 copies per µl.

The stool positivity for enterovirus was connected to more than two-fold increase in the frequency of enterovirus in blood (examples of enterovirus in blood following gut infection are shown in **Figure 1**). This may be an indication that viraemia arose from the strain present in the gut, or a reflection of a general propensity towards enterovirus infection both in the gut and blood. Enterovirus genotyping would be required to answer the question on identity of the strains: this will be pursued further, but it should be noted that discrimination of the enterovirus genotype present in blood is technically extremely difficult and many obstacles have to be overcome before the low-quantity enterovirus RNA can be genotyped.

Interestingly, only 25% enterovirus-positive blood samples had their respective concomitant stool also positive. There may be several explanations; however, neither of them can be tested in the present dataset. Firstly, the primary replication of the virus may have taken place elsewhere than in the gut (e.g. tonsils, nasal cavity), or may have been too short to be covered by the monthly intervals between stool samples. Secondly, the presence of virus in blood can be an indication of viral persistence long after the primary replication. Lastly, the presence (and quantity) of virus RNA in blood may vary with time, so that a single sample can ascertain only a fraction of subjects who have the virus replicating in their organs.

### Strength and weaknesses of the present work

The frequency of enterovirus in blood is considerably higher in our study as compared to what other groups observed in nondiabetic individuals (e.g. [5], [6], [33], [36]), rendering our study higher power to detect differences among *IFIH1* genotypes. The increased frequency may be a combination of epidemiological reasons (e.g. the age composition of the observed cohorts, difference between the Norwegian and other populations) and methodological aspects: in contrast to other studies, we used the blood cell pack as the source of nucleic acid - this may be an effective strategy given that the location of virus within the blood fractions has not been thoroughly studied in various phases of the infection. Interestingly, closely related *Human parechovirus* detected using a similar nested technique in a subset of the present blood samples was observed far less often (5/234 samples, 2.1%, 95% CI 0.94%–4.90%, data not shown), so that the possibility of epidemiological reasons still remains open.

A limitation with the current study is that each individual was represented by a single blood sample only. Also the relation between enterovirus RNA in blood and in stool could be better assessed with more frequent stool collection – yet our monthly sampling schedule is equally or more frequent as in other ongoing studies on diabetes (reviewed by [35]), being a compromise for the sake of the parents' compliance. Future large scale studies investigating the potential relation between enterovirus RNA in the blood and risk of islet autoimmunity or T1D should probably include genotyping data for polymorphisms in the *IFIH1* gene to hopefully arrive at a better understanding of the interrelationship among these factors.

### Conclusion

We found that the common *IFIH1* SNP rs1990760 was significantly associated with frequency of enterovirus RNA in blood of healthy children, and that this association was independent of enterovirus RNA in the faeces. This may help explain the mechanisms of *IFIH1* association with T1D.

## References

[1] Tauriainen S, Oikarinen S, Oikarinen M, Hyoty H (2010) Enteroviruses in the pathogenesis of type 1 diabetes. Semin Immunopathol 10.1007/s00281-010-0207-y 20424841

[2] Oikarinen M, Tauriainen S, Honkanen T, Vuori K, Karhunen P, et al (2008) Analysis of pancreas tissue in a child positive for islet cell antibodies. Diabetologia 51: 1796–1802. 18696046 10.1007/s00125-008-1107-8

[3] Oikarinen M, Tauriainen S, Oikarinen S, Honkanen T, Collin P, et al (2012) Type 1 diabetes is associated with enterovirus infection in gut mucosa. Diabetes 61: 687–691. 22315304 10.2337/db11-1157 PMC3282798

[4] Richardson SJ, Willcox A, Bone AJ, Foulis AK, Morgan NG (2009) The prevalence of enteroviral capsid protein vp1 immunostaining in pancreatic islets in human type 1 diabetes. Diabetologia 52: 1143–1151. 19266182 10.1007/s00125-009-1276-0

[5] Lonnrot M, Salminen K, Knip M, Savola K, Kulmala P, et al (2000) Enterovirus RNA in serum is a risk factor for beta-cell autoimmunity and clinical type 1 diabetes: a prospective study. Childhood Diabetes in Finland (DiMe) Study Group. J Med Virol 61: 214–220. 10797377

[6] Oikarinen S, Martiskainen M, Tauriainen S, Huhtala H, Ilonen J, et al (2011) Enterovirus RNA in Blood Is Linked to the Development of Type 1 Diabetes. Diabetes 60: 276–279. 20943747 10.2337/db10-0186 PMC3012181

[7] Sadeharju K, Hamalainen AM, Knip M, Lonnrot M, Koskela P, et al (2003) Enterovirus infections as a risk factor for type I diabetes: virus analyses in a dietary intervention trial. Clin Exp Immunol 132: 271–277. 12699416 10.1046/j.1365-2249.2003.02147.x PMC1808709

[8] Salminen K, Sadeharju K, Lonnrot M, Vahasalo P, Kupila A, et al (2003) Enterovirus infections are associated with the induction of beta-cell autoimmunity in a prospective birth cohort study. J Med Virol 69: 91–98. 12436483 10.1002/jmv.10260

[9] Fuchtenbusch M, Irnstetter A, Jager G, Ziegler AG (2001) No evidence for an association of coxsackie virus infections during pregnancy and early childhood with development of islet autoantibodies in offspring of mothers or fathers with type 1 diabetes. J Autoimmun 17: 333–340. 11771958 10.1006/jaut.2001.0550

[10] Graves PM, Rotbart HA, Nix WA, Pallansch MA, Erlich HA, et al (2003) Prospective study of enteroviral infections and development of beta-cell autoimmunity. Diabetes autoimmunity study in the young (DAISY). Diabetes Res Clin Pract 59: 51–61. 12482642 10.1016/s0168-8227(02)00198-5

[11] Kato H, Takeuchi O, Sato S, Yoneyama M, Yamamoto M, et al (2006) Differential roles of MDA5 and RIG-I helicases in the recognition of RNA viruses. Nature 441: 101–105. 16625202 10.1038/nature04734

[12] Gitlin L, Barchet W, Gilfillan S, Cella M, Beutler B, et al (2006) Essential role of mda-5 in type I IFN responses to polyriboinosinic:polyribocytidylic acid and encephalomyocarditis picornavirus. Proc Natl Acad Sci U S A 103: 8459–8464. 16714379 10.1073/pnas.0603082103 PMC1464000

[13] Chistiakov DA (2010) Interferon induced with helicase C domain 1 (IFIH1) and virus-induced autoimmunity: a review. Viral Immunol 23: 3–15. 20121398 10.1089/vim.2009.0071

[14] Smyth DJ, Cooper JD, Bailey R, Field S, Burren O, et al (2006) A genome-wide association study of nonsynonymous SNPs identifies a type 1 diabetes locus in the interferon-induced helicase (IFIH1) region. Nat Genet 38: 617–619. 16699517 10.1038/ng1800

[15] Enevold C, Oturai AB, Sorensen PS, Ryder LP, Koch-Henriksen N, et al (2009) Multiple sclerosis and polymorphisms of innate pattern recognition receptors TLR1-10, NOD1-2, DDX58, and IFIH1. J Neuroimmunol 212: 125–131. 19450885 10.1016/j.jneuroim.2009.04.008

[16] Martinez A, Santiago JL, Cenit MC, de Las Heras V, de la Calle H, et al (2008) IFIH1-GCA-KCNH7 locus: influence on multiple sclerosis risk. Eur J Hum Genet 16: 861–864. 18285833 10.1038/ejhg.2008.16

[17] Sutherland A, Davies J, Owen CJ, Vaikkakara S, Walker C, et al (2007) Genomic polymorphism at the interferon-induced helicase (IFIH1) locus contributes to Graves' disease susceptibility. J Clin Endocrinol Metab 92: 3338–3341. 17535987 10.1210/jc.2007-0173 PMC6952273

[18] Nejentsev S, Walker N, Riches D, Egholm M, Todd JA (2009) Rare variants of IFIH1, a gene implicated in antiviral responses, protect against type 1 diabetes. Science 324: 387–389. 19264985 10.1126/science.1167728 PMC2707798

[19] Downes K, Pekalski M, Angus KL, Hardy M, Nutland S, et al (2010) Reduced expression of IFIH1 is protective for type 1 diabetes. PLoS One 5. 10.1371/journal.pone.0012646 PMC2936573 20844740

[20] Liu S, Wang H, Jin Y, Podolsky R, Linga Reddy MV, et al (2008) The IFIH1 Polymorphisms Are Significantly Associated with Type 1 Diabetes and IFIH1 Gene Expression in Peripheral Blood Mononuclear Cells. Hum Mol Genet 10.1093/hmg/ddn342 PMC2638779 18927125

[21] Shigemoto T, Kageyama M, Hirai R, Zheng J, Yoneyama M, et al (2009) Identification of loss of function mutations in human genes encoding RIG-I and MDA5: implications for resistance to type I diabetes. J Biol Chem 284: 13348–13354. 19324880 10.1074/jbc.M809449200 PMC2679434

[22] Chistiakov DA, Voronova NV, Savost'Anov KV, Turakulov RI (2010) Loss-of-function mutations E6 27X and I923V of IFIH1 are associated with lower poly(I∶C)-induced interferon-beta production in peripheral blood mononuclear cells of type 1 diabetes patients. Hum Immunol 71: 1128–1134. 20736039 10.1016/j.humimm.2010.08.005

[23] Witsø E, Tapia G, Cinek O, Pociot FM, Stene LC, et al (2011) Polymorphisms in the innate immune IFIH1 gene, frequency of enterovirus in monthly fecal samples during infancy, and islet autoimmunity. PLoS One 6: e27781. 22110759 10.1371/journal.pone.0027781 PMC3215739

[24] Stene LC, Witso E, Torjesen PA, Rasmussen T, Magnus P, et al (2007) Islet autoantibody development during follow-up of high-risk children from the general Norwegian population from three months of age: Design and early results from the MIDIA study. Journal of Autoimmunity 29: 44–51. 17560077 10.1016/j.jaut.2007.04.003

[25] Witsø E, Cinek O, Tapia G, Rasmussen T, Stene LC, et al (2012) HLA-DRB1-DQA1-DQB1 genotype and frequency of enterovirus in longitudinal monthly fecal samples from healthy infants. Viral Immunol 25. 10.1089/vim.2012.0001 22691100

[26] Verstrepen WA, Kuhn S, Kockx MM, Van de Vyvere ME, Mertens AH (2001) Rapid detection of enterovirus RNA in cerebrospinal fluid specimens with a novel single-tube real-time reverse transcription-PCR assay. J Clin Microbiol 39: 4093–4096. 11682535 10.1128/JCM.39.11.4093-4096.2001 PMC88492

[27] Garcia-Closas M, Lubin JH (1999) Power and sample size calculations in case-control studies of gene-environment interactions: comments on different approaches. Am J Epidemiol 149: 689–692. 10206617 10.1093/oxfordjournals.aje.a009876

[28] Comings DE, MacMurray JP (2000) Molecular heterosis: a review. Mol Genet Metab 71: 19–31. 11001792 10.1006/mgme.2000.3015

[29] Aminkeng F, Van Autreve JE, Weets I, Quartier E, Van Schravendijk C, et al (2009) IFIH1 gene polymorphisms in type 1 diabetes: genetic association analysis and genotype-phenotype correlation in the Belgian population. Hum Immunol 70: 706–710. 19539001 10.1016/j.humimm.2009.06.013

[30] Zouk H, Marchand L, Polychronakos C (2010) Study of transcriptional effects in Cis at the IFIH1 locus. PLoS One 5: e11564. 20644636 10.1371/journal.pone.0011564 PMC2903489

[31] Berke IC, Modis Y (2012) MDA5 cooperatively forms dimers and ATP-sensitive filaments upon binding double-stranded RNA. EMBO J 31: 1714–1726. 22314235 10.1038/emboj.2012.19 PMC3321199

[32] Cui S, Eisenacher K, Kirchhofer A, Brzozka K, Lammens A, et al (2008) The C-terminal regulatory domain is the RNA 5′-triphosphate sensor of RIG-I. Mol Cell 29: 169–179. 18243112 10.1016/j.molcel.2007.10.032

[33] Schulte BM, Bakkers J, Lanke KH, Melchers WJ, Westerlaken C, et al (2010) Detection of enterovirus RNA in peripheral blood mononuclear cells of type 1 diabetic patients beyond the stage of acute infection. Viral Immunol 23: 99–104. 20121407 10.1089/vim.2009.0072

[34] Pallansch MA, Roos RP (1997) Enteroviruses: Polioviruses, Coxsackieviruses, Echoviruses, and Newer Enteroviruses. In: Knipe DM, Howley PM, editors. Fields' virology. Philadelphia: Lippincott Williams & Wilkins. pp 723–775.

[35] Stene LC, Rewers M (2012) Immunology in the clinic review series; focus on type 1 diabetes and viruses: the enterovirus link to type 1 diabetes: critical review of human studies. Clin Exp Immunol 168: 12–23. 22385232 10.1111/j.1365-2249.2011.04555.x PMC3390488

[36] Moya-Suri V, Schlosser M, Zimmermann K, Rjasanowski I, Gurtler L, et al (2005) Enterovirus RNA sequences in sera of schoolchildren in the general population and their association with type 1-diabetes-associated autoantibodies. J Med Microbiol 54: 879–883. 16091441 10.1099/jmm.0.46015-0

[37] Jermendy A, Szatmari I, Laine AP, Lukacs K, Horvath KH, et al (2010) The interferon-induced helicase IFIH1 Ala946Thr polymorphism is associated with type 1 diabetes in both the high-incidence Finnish and the medium-incidence Hungarian populations. Diabetologia 53: 98–102. 19841890 10.1007/s00125-009-1561-y

[38] Gaunt TR, Rodriguez S, Day IN (2007) Cubic exact solutions for the estimation of pairwise haplotype frequencies: implications for linkage disequilibrium analyses and a web tool ‘CubeX’. BMC Bioinformatics 8: 428. 17980034 10.1186/1471-2105-8-428 PMC2180187

